# Determination of natural progesterone levels in bovine serum using gas chromatography coupled with mass spectrometry

**DOI:** 10.2478/jvetres-2025-0071

**Published:** 2025-12-16

**Authors:** Iwona Matraszek-Żuchowska, Alicja Kłopot, Paulina Zdonek, Beata Korycińska, Justyna Grzelak

**Affiliations:** Department of Food and Feed Chemical Research, National Veterinary Research Institute, 24-100 Puławy, Poland

**Keywords:** bovine serum, GC-MS, natural hormones, oestrous cycle, progesterone

## Abstract

**Introduction:**

Progesterone, a steroidal female sex hormone, can be used for anabolic purposes in cattle. Physiological levels of progesterone in animals are highly variable, and systematic studies of them in various matrices are limited. The purpose of the present study was to apply a developed and validated method routinely used for the determination of the natural hormones 17β-oestradiol and 17β-testosterone in serum to estimate natural cattle progesterone levels. The analytes were taken from serum samples collected from cattle as part of the Polish National Residue Control Plan.

**Material and Methods:**

A total of 1,537 bovine serum samples were analysed: 895 from females and 642 from males. Progesterone was extracted from serum with a mixture of tert-butyl methyl ether and petroleum ether, the extract was derivatised and the analyte was determined by gas chromatography coupled with mass spectrometry.

**Results:**

The basic validation parameters specified for the method, namely repeatability, reproducibility and apparent recovery, indicated it to be useful for the intended purpose. The natural presence of progesterone was detected in 679 samples from females and in 375 samples from males. Progesterone in females was measured in the range of 0.001–496.059 μg L^−1^ (in 61% of samples it was below 1 μg L^−1^ and in 32% it was in the range of 1–7 μg L^−1^, which are the physiological ranges of phases of the reproductive cycle) and progesterone in males was assayed in the range of 0.001–31.792 μg L^−1^.

**Conclusion:**

The results obtained may serve as a statistical basis for further research.

## Introduction

Natural hormones are chemical compounds produced by the body that regulate various life processes, from metabolism, governed by thyroid hormones and insulin, to growth and development, activated by cortisol, melatonin and sex hormones. Among the natural sex hormones, oestrogens and progesterone (pregn-4-ene-3,20-dione, P4 or lutein) are key to the menstrual cycle and pregnancy, while testosterone plays an important role in both sexes, affecting libido, energy and muscle strength and performance.

Progesterone is one of the most important natural sex hormones in females because of its role in the body. It is produced mainly by the cells of the corpus luteum of the ovary during the luteal phase of the menstrual cycle and in early pregnancy, and by the placenta in the later stages of pregnancy (4, 20, 22). This hormone is in the progestogen group, which are steroid hormones. An upstream precursor of P4, as it is of many other important steroid hormones, is cholesterol, which is converted into pregnenolone, its direct precursor.

The main function of P4 is to prepare the uterus for embryo implantation in its mucous membrane and to protect it throughout pregnancy (4, 20, 22). The effects of P4 on the reproductive organs include facilitating ovulation, stimulating growth or inhibiting an excess of it in the endometrium, and causing cyclical changes in the epithelium of the reproductive organs. It also acts as a neurotransmitter, transporting information between nerve cells in the brain, especially in areas responsible for regulating sexual behaviour and sex drive (22).

Progesterone works synergistically with oestrogens, thereby stimulating lactation. In human medicine, this hormone is used prophylactically in women at risk of miscarriage, with corpus luteum insufficiency, with pregnancy poisoning, in the treatment of infertility, in menstrual cycle disorders resulting from endogenous hormone deficiency, and in postmenopausal hormone replacement therapy for protective purposes. Although P4 is a female sex hormone, it also plays an important role in the bodies of males. It inhibits the conversion of testosterone to dihydrotestosterone, excessive levels of which can lead to serious diseases such as prostate cancer. In men, P4 is produced by the testes and adrenal glands in much smaller combined amounts than the combined amounts of P4 produced by the ovaries and adrenal glands in women.

As human medicine uses P4, so does veterinary medicine, mainly in reproduction and embryogenesis, where it is applied in therapeutic and preventive treatment. Either P4 alone or the hormone in combination with 17β-oestradiol (17β-E2) may be administered in the form of implants to farm animals intended for slaughter in order to improve muscle growth and feed conversion, *i.e*. for anabolic growth promotion. An example of this are implants containing P4 and oestradiol benzoate (Implus-S or Synovex C) administered to breeding cattle on farms in the United States, Australia, and other countries. The duration and effectiveness of P4 as an anabolic agent can be prolonged by converting it into a synthetic derivative, medroxyprogesterone acetate (MPA). Medroxyprogesterone acetate is an acetylgestagen, as are megestrol acetate (MGA), chlormadinone acetate (CMA), melengestrol acetate (MLGA), flugestone acetate and delmadinone acetate. These androgenic steroid hormones with anabolic properties are compounds formed as a result of hydroxylation and esterification of P4 at the C17 position and the introduction of a double bond between the C6 and C7 carbon atoms and a methyl group or chlorine atom on the carbon atom 6 of the four-ring molecule. In human medicine, synthetic acetylgestagens are mainly used in gynaecology; in veterinary medicine, they may also be administered for therapeutic purposes. Therapy is not the only use of them in veterinary practice: outside the European Union, melengestrol acetate may be used as a feed additive in preparations for heifers intended for breeding and slaughter (3).

Natural steroid hormones are present in almost all biological matrices in different animal species. Their complex interrelationships complicate their monitoring and the interpretation of residue testing results. In European Union countries, the use of hormonal compounds in the fattening of animals intended for slaughter has been prohibited since 1988 (8, 15); therefore, residue testing is necessary in the bloc. In connection with the recommendation to test natural sex hormone levels in animal material on the farm and at the slaughterhouse, only for 17β-E2 and 17β-testosterone (17β-T) in cattle serum have maximum concentration values been established. They were set on the basis of scientific research for the sex and age of the animal, which are taken into account when interpreting test results. These compounds are specifically mentioned in the technical guidance on minimum method performance requirements (MMPR) issued by the EU Reference Laboratories (EURLs) supported by the European Commission (11). The third natural steroid hormone considered in the EURL Reflection Paper on natural growth-promoting substances in biological samples issued by the Wageningen Food Safety Research EURL (WFSR) in Wageningen in the Netherlands is P4, and the guidance is to replace it with synthetic analogues in monitoring studies (2). However, unpublished scientific studies conducted at Wageningen indicated variable levels of P4 concentration in biological matrices such as urine (2).

Currently, laboratories in the European Union generally attempt to detect synthetic P4 derivatives in kidney fat tissue and muscle samples (MPA, MGA, CMA and MLGA) and feed samples (MPA) as part of monitoring and individual consumer studies (5). The availability of serum samples for testing for the natural hormones 17β-E2 and 17β-T taken from Polish animals as part of the National Residue Control Plan and the lack of a statistical database for P4 in serum prompted the undertaking of research to estimate the natural concentrations of P4 in this matrix for cattle populations of both sexes and different ages, and thus to create a statistical database that could be used in the further stage of the legislated monitoring of hormone residues (13, 16).

## Material and Methods

### Serum samples

The main part of the study used cattle serum samples collected in the years 2017–2025 by the Veterinary Inspectorate for testing as part of the risk-based National Residue Control Plan in Poland. The samples were taken from animals living on farms and animals delivered to the slaughterhouse. A total of 1,537 serum samples were tested during more than eight years of research. Serum samples were taken from 895 heifers and cows between 2 weeks and 204 months of age and from 642 bulls and steers between 3 weeks and 120 months of age. Young cattle up to 18 months of age were sampled, in which risking the use of hormones for anabolic purposes may have a basis, since the animals are intended for breeding and slaughter, but also older cattle were sampled, which was determined mainly by the availability of animals on farms. The animals of both sexes were grouped by age according to the European Food Safety Authority classification for monitoring studies conducted in the EU. This distribution includes beef and dairy calves up to 12 months of age, calves, young bulls, and heifers aged 12 to 24 months, and breeding bulls, dairy cows, beef cattle, and steers older than 2 years of age. Because of the special importance of P4 in reproduction for female cattle, subdivision into an age group below 6 months of age and a group between 6 and 12 months of age was specified, for analysis of animals grouped in the period when the animals mature and show sexual activity.

Sex and age data were taken from a test order generated electronically, but additional breed, physiological condition and feeding regime information was not available. The test order classified the cattle examined primarily as meat-producing, but also as mixed-breed and dairy cattle. The protocols did not contain detailed information on the cattle breeds, so it is likely that the cattle were Holstein-Friesian, Simmental and Charolais, because these breeds are raised on Polish farms.

Blood samples from cattle were collected in tubes without anticoagulants to obtain a clot and transported to the laboratory at a temperature of 2–8°C. In the laboratory, the blood was centrifuged to separate the serum. The serum was either tested immediately or stored frozen (<–18°C) until testing to ensure the stability of the analytes in the matrix. The samples were tested within 30 calendar days of arrival at the laboratory in accordance with the arrangements for monitoring tests.

### Reagents and chemicals

All chemicals were of high grade of purity. Petroleum ether, tert-butyl methyl ether and methanol (residue grade) were purchased from J.T. Baker (Deventer, the Netherlands), and isooctane (GC grade) and heptafluorobutyric anhydride (HFBA) were obtained from Sigma-Aldrich (Steinheim, Germany).

Standards of P4 (C_21_H_30_O_2_, molecular weight (m.w.) 314.46 Da, CAS 57-83-0; 98% purity) and 17β-testosterone-D2 (the deuterated analogue of 17β-T used as internal standard, 17β-T-D2, C_19_H_22_D_2_O_2_, m.w. 290.44 Da, CAS 204244-83-7; >95% purity) were obtained from Dr Ehrenstorfer (Augsburg, Germany), and Wageningen Food Safety Research (Wageningen, the Netherlands). The P4 standard was kept at room temperature, and the labelled standard was stored at 2–8°C as recommended in their accompanying certificates.

Primary standard stock solutions were prepared in methanol at concentrations of 1 mg mL^−1^ and 10 μg mL^−1^ and were stored at <–18°C for no longer than one year. Working solutions were obtained by tenfold dilution of primary standard solutions to the concentration of 1 μg mL^−1^ in methanol and were stored at a temperature of at 2–8°C for not longer than six months.

### Sample preparation

Hormones were obtained from serum samples as guided in the literature (31, 32). In brief, the hormones were extracted from serum samples using a mixture of tert-butyl methyl ether/petroleum ether (30:70, v/v). After phase separation, the contents of the tubes were centrifuged and frozen at <–18°C for 1.5–2 h. The organic phase was then decanted and evaporated to dryness under a gentle stream of nitrogen. The residue was derivatised with HFBA before GC-MS. For each analytical series, a calibration curve was prepared on standards within a specific range of analyte concentrations. The P4 determined by GC-MS was quantified using the internal standard technique. In each analytical series, samples spiked with the analyte to a specific concentration were tested in parallel to verify the quality of the results.

### GC-MS analysis

Serum samples were analysed using gas chromatography coupled with mass spectrometry (GC-MS), as described previously (31, 32). The published method is accredited for the determination of the natural sex hormones 17β-T and 17β-E2 in serum and has been positively verified in proficiency tests and inter-laboratory comparisons. A 6890N GC chromatograph with an Agilent 5973 hyperbolic quadrupole Mass Selective Detector and ChemStation software (all from Agilent Technologies, Wilmington, DE, USA) was used for the analysis. Chromatographic separation of P4 and 17β-T-D2 was achieved on a non-polar HP-5MS (mass-spectrometry-compatible) capillary column of 30 m length 0.25 mm internal diameter and 0.25 μm film thickness using a 0.9 mL min^−1^ constant flow of helium. The samples were injected at 2 μL volume in pulsed splitless mode at 250°C. The oven temperature was held at 120°C for 2 min, increased by 7°C per min to 280°C and held for 2 min, increased by 30°C to 310°C and also held for 2 min. The injection port, MS source and quadrupole temperatures were set at 250, 230 and 150°C, respectively. The GC-MS apparatus was operated in positive electron impact ionisation mode at 70 eV with selected ion monitoring. The GC-MS diagnostic ions (m/z) for detection and quantification of P4 and the internal standard of 17β-T-D2 for were 510 and 682, respectively.

### Validation study

For the method’s validation, basic performance parameters were determined, namely linearity, repeatability, reproducibility, apparent recovery and decision limit (7, 12). The instrumental linearity was assessed based on standard calibration curves of standard working solutions of P4 drawn through seven points and adjusted to the spiking range of the serum samples, which corresponded to 0.000, 0.100, 0.200, 0.500, 1.000, 2.000 and 6.000 μg L^−1^. In order to determine the apparent recovery, precision (repeatability) and reproducibility and to assess the linearity of matrix-matched calibration curves, blank serum samples spiked with P4 to the concentration levels of 0.500 μg L^−1^, 1.000 μg L^−1^ and 2.000 μg L^−1^ were analysed. The spiked samples were tested in three series, each with six replicates at each spiking level. For both calibration curves (standard and matrix-matched), regression parameters were calculated. In the analysed samples, concentration calculations were based on the standard calibration curves prepared in the current series of studies and those with the addition of internal standard. When the P4 concentration exceeded the range of the standard curve adopted in validation, an extended standard curve was created by extrapolation, enabling the reliable calculation of the concentration in the sample. The values of detection capability (CCβ) and decision limit (CCa) were determined on the basis of a matrix-matched calibration curve. The mathematical formulae stipulated in the ISO 11843-2 standard were used to define these quantities: the CCa was calculated as the corresponding concentration at the y-intercept of the calibration line plus 2.33 times the standard deviation of the within-laboratory reproducibility of the intercept, while the CCβ was calculated as the corresponding concentration at the decision limit plus 1.64 times the standard deviation of the within-laboratory reproducibility of the intercept (19). The MUkit (Measurement Uncertainty kit) software tool v. 3.0 was implemented for the estimation of measurement uncertainty using laboratory validation quality data (17). The software is mainly based on the Nordtest 537 Technical Report (17).

## Results

A summary of the performance of the method for P4 detection is presented in Table 1.

**Table 1.** Method parameters for P4 detection in bovine serum samples (n = 18)

Parameter	Spiking level (μg L^−1^)	
Mean concentration (μg L^−1^) / apparent recovery (%)	0.500	0.433 / 86.5
	1.000	0.893 / 89.3
	2.000	1.778 / 88.9
Repeatability (s_r_, μg L^−1^) / RSD (%)	0.500	0.056 / 12.8
	1.000	0.111 / 12.4
	2.000	0.333 / 18.7
Within-lab reproducibility (s_R_, μg L^−1^) / RSD (%)	0.500	0.066 / 15.2
	1.000	0.193 / 21.6
	2.000	0.366 / 20.6
Decision limit (CCa, μg L^−1^)		0.058
Detection capability (CCβ, μg L^−1^)		0.100
Expanded measurement uncertainty (U, k = 2, %)	0.500	47
	1.000	50
	2.000	55
Standard calibration curve		
Slope ± s_b_		3.2134 ± 0.7344
y-intercept ± s_a_		0.1394 ± 0.0642
Correlation coefficient		0.9999
Standard error		0.1169
Matrix-matched calibration curve		
Slope ± s_b_		2.8158 ± 0.0242
y-intercept ± s_a_		-0.0952 ± 0.7603
Correlation coefficient		0.9999
Standard error		0.0951

1 RSD – relative standard deviation; s_r_ – standard deviation under repeatability conditions; s_R_ – standard deviation under reproducibility conditions; s_a_ – standard deviation of slope; s_b_ – standard deviation of intercept

The linear regression parameters for the standard and matrix-matched calibration curves of P4 were correct over the entire assumed concentration range. The calculated correlation coefficients for the plotted curves were greater than 0.98, as shown in Table 1. Apparent recovery of P4 from serum at every spiking level of validation exceeded 80%, with the relative standard deviation not exceeding 20% and being less than 25%under reproducibility conditions. The calculated CCβ and CCa values as presented in Table 1 were 0.100 and 0.058 μg L^−1^, respectively and were approximately five and ten times lower than the lowest level of validation and also than the lowest MMPR (0.5 μg L^−1^) set for 17β-T in serum from female cows younger than 18 months. The determined values of expanded measurement uncertainty expressed as percentages were up to 55% depending on the spiking level.

Summary and statistics of the number of serum samples from cattle tested for P4 content are given in Tables 2 and 3.

**Table 2.** Detection of progesterone (P4) by cattle sex and age in the earlier part of the investigation

		Age (months)	Year of sampling				
			2017	2018	2019	2020	2021
Male cattle	Sample size		122	124	117	59	52
	Number tested / with detected P4	>12	22 / 7	35 / 26	26 / 26	6 / 4	7 / 2
		<12 to ≤24	94 / 36	78/ 67	69 / 60	42 / 24	38 / 19
		>24	5 / 4	8 / 7	15 / 13	10 / 7	7 / 4
		not specified	1 / 1	3 / 1	7 / 0	1 / 0	0 / 0
Female cattle	Sample size		132	146	140	165	130
	Number tested / with detected P4	≤6	20 / 2	37 / 25	15 / 14	8 / 6	8 / 4
		>6 to ≤12	12 / 6	11 / 10	17 / 17	9 / 5	4 / 1
		≤12	32 / 8	48 / 35	32 / 31	17 /11	12 / 5
		>12 to ≤24	64 / 32	60 / 58	41 / 40	60 / 48	38 / 28
		>2	35 / 21	36 / 36	67 / 65	88 / 78	80 / 65
		not specified	1 / 0	2 / 2	0 / 0	0 / 0	0 / 0
Male and female cattle	Combined all-age sample size		254	270	257	224	182
Male cattle	All-age number tested with detected P4		48	101	99	35	25
	Percentage		39	81	85	59	48
Female cattle	All-age number tested with detected P4		61	131	136	137	98
	Percentage		46	90	97	83	75
Male and female cattle	Combined all-age number tested with detected P4		109	232	235	172	123
	Percentage		43	86	91	77	68

**Table 3.** Detection of progesterone (P4) by cattle sex and age in the later part of the investigation and a summary of the entire research period

		Age (months)	Year of sampling				
			2022	2023	2024	2025	2017–2021
Male cattle	Sample size		79	34	43	12	642
	Number tested / with detected P4	≤12	6 / 0	4 / 1	3 / 2	0 / 0	109 / 68
		>12 to ≤24	61 / 20	21/ 5	28 / 10	7 / 6	438 / 247
		>24	12 / 6	8 / 3	10 / 7	5 / 5	80 / 56
		not specified	0 / 0	1 / 1	2 / 1	0 / 0	15 / 4
Female cattle	Sample size		95	55	27	5	895
	Number tested / with detected P4	≤6	0 / 0	2 / 0	1 / 1	1 / 1	92 / 53
		>6 to ≤12	3 / 0	4 / 0	2 / 0	0 / 0	62 / 39
		≤12	3 / 0	6 / 0	3 / 1	1 / 1	154 / 92
		>12 to ≤24	38 / 22	24 / 17	8 / 6	3 / 3	336 / 254
		>24 months	54 / 31	25 / 21	14 / 11	1 / 1	400 / 329
		not specified	0 / 0	0 / 0	2 / 2	0 / 0	5 / 4
Male and female cattle	Combined all-age sample size		174	89	70	17	1,537
Male cattle	All-age number tested with detected P4		26	10	20	11	375
	Percentage		33	29	47	92	57
Female cattle	All-age number tested with detected P4		53	38	20	5	679
	Percentage		56	69	74	100	77
Male and female cattle	Combined all-age number tested with detected P4		79	48	40	16	1,054
	Percentage		45	54	57	94	68

Progesterone was detected in 375 serum samples from males of the various age groups mentioned above, representing 57% of the samples tested. In the group of male cattle up to 12 months of age, the percentage of serum samples containing P4 was 62%, in the group aged 12 to 24 months it was 56%, and for male cattle aged over 2 years it was 70%. In the case of female cattle, P4 was detected in 679 serum samples from animals of the same age range as males, representing 77% of the samples tested. In the group of female cattle up to 12 months of age, the percentage of serum samples containing P4 was 60%, in the group aged 12 to 24 months it was 76%, and for female cattle aged over 2 years it was 82%. Among the sub-yearling females, individuals up to 6 months of age were analysed separately from those older because they mature and reach sexual maturity between 7 and 9 months of age. In the group aged up to 6 months, P4 was detected in 58% of animals, and in the group of females aged 6 to 12 in 63%. In total, P4 was detected in 1,054 serum samples from male and female young and adult cattle, which was 68% of all samples tested.

**Fig. 1.**
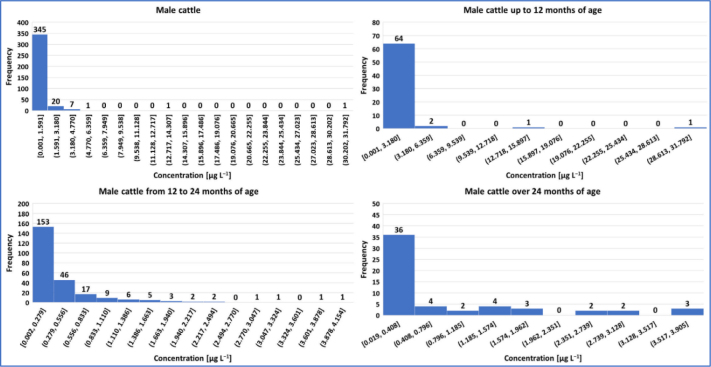
Histogram of progesterone concentrations in male cattle

**Fig. 2.**
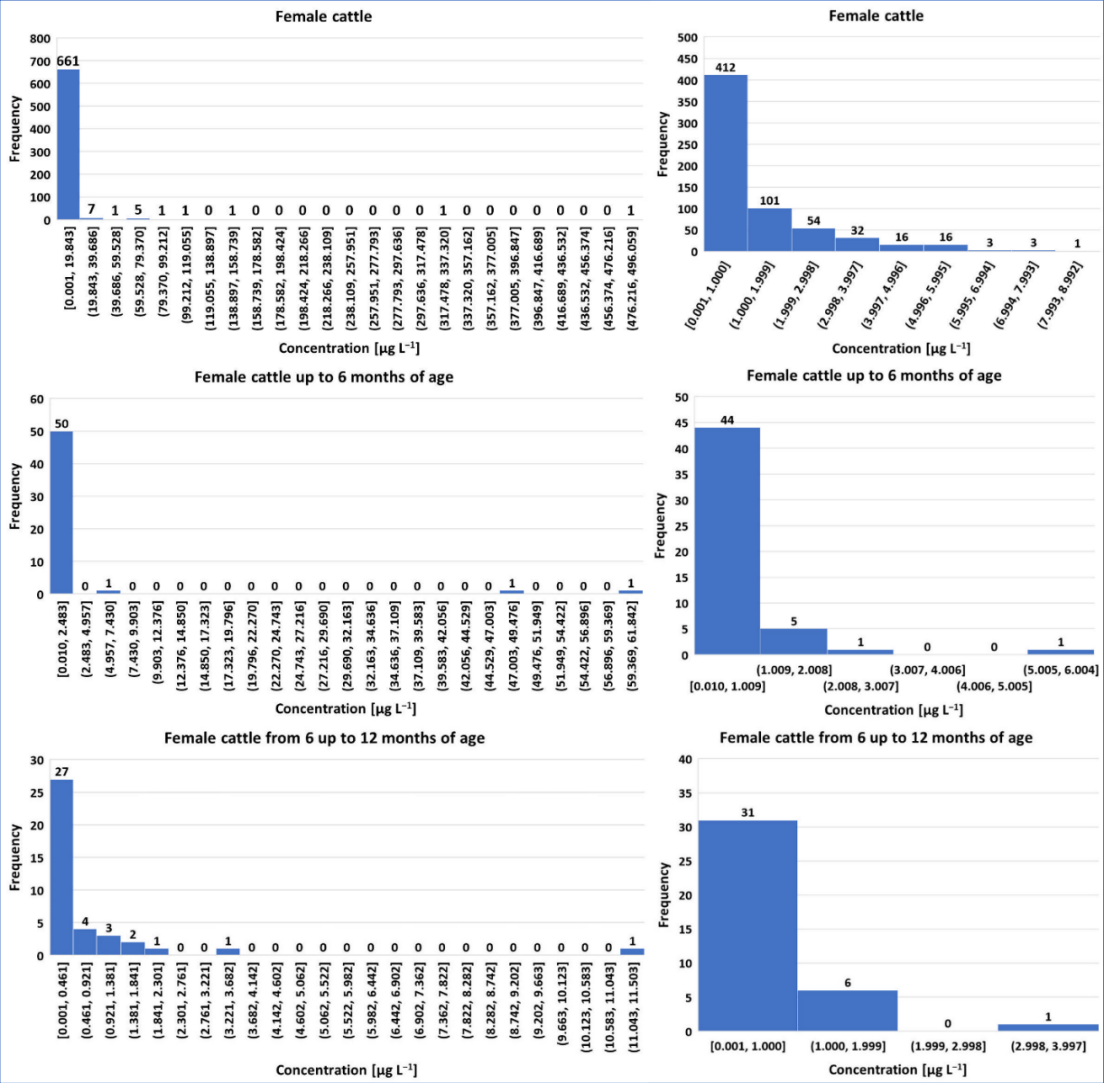
Histogram of progesterone concentrations in female cattle. The charts on the left present concentration data for specific age groups (including the entire cows population); the histograms on the right side show progesterone concentrations that may indicate the current oestrous cycle phase

**Fig. 3.**
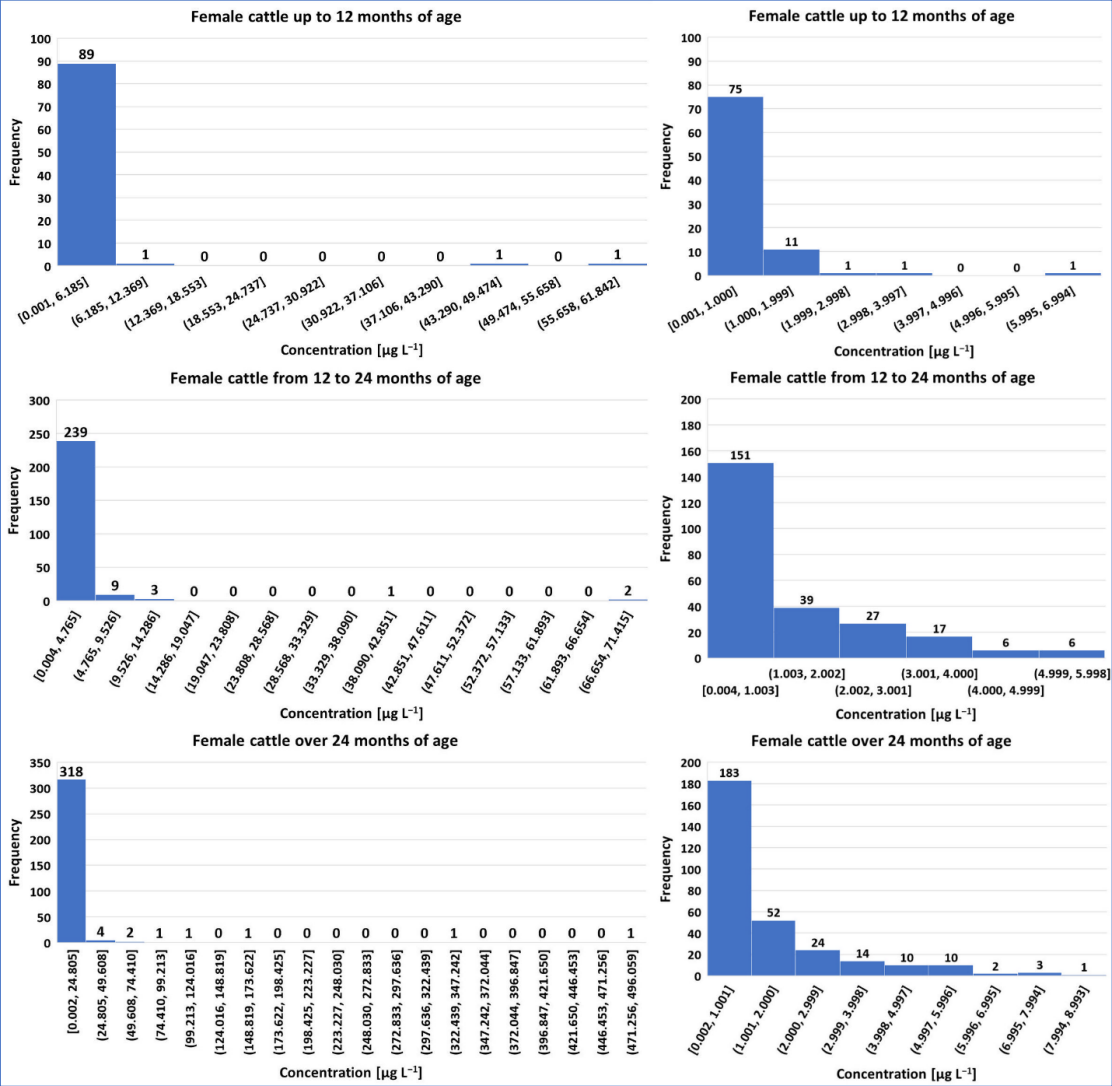
Histogram of progesterone concentrations in female cattle. The charts on the left present concentration data for specific age groups; the histograms on the right side show progesterone concentrations that may indicate the current oestrous cycle phase (continuation of Fig. 2)

Summary statistical data for the concentrations of P4 in bovine animals are presented in Table 4, and the graphical presentation of the results in the form of histograms is presented in Figs 1, 2 and 3.

**Table 4.** Serum progesterone (P4) concentration levels in the studied cattle population, and levels in females that may indicate the current oestrous cycle phase

	Age (months)		Concentration (μg L^−1^)							Number of results below decision limit	Percentage of results below decision limit
			Minimum	Maximum	Mean	Median	Standard deviation	95^th^Centile	99^th^Centile		
Male cattle	≤12		0.001	31.792	1.170	0.174	4.187	4.038	19.227	16	24
	>12 to ≤24		0.002	4.154	0.385	0.138	0.598	1.572	3.044	65	26
	>24		0.019	3.905	0.727	0.172	1.042	3.138	3.804	7	13
	All ages combined		0.001	31.792	0.575	0.155	1.905	2.169	4.310	90	24
Female cattle	≤6		0.010	61.842	2.541	0.216	10.615	3.640	54.755	10	19
	>6 to ≤12		0.001	11.503	0.791	0.206	1.895	2.038	8.442	5	13
	≤12		0.001	61.842	1.799	0.211	8.164	2.689	49.439	15	16
	>12 to ≤24		0.004	71.415	2.031	0.659	6.706	5.108	23.866	28	11
	>24		0.002	496.059	6.348	0.720	35.245	13.667	109.185	35	11
	All ages combined		0.001	496.059	4.083	0.622	25.130	8.542	68.112	78	11
Female cattle	≤6	P4 conc. <1 (oestrus)	0.010	0.996	0.240	0.108		0.717	0.968		
		P4 conc. >1 (ovulation)	1.011	5.998	2.009	1.151		4.819	5.762		
		P4 conc. ≥3 and ≤7 (midluteal period)	5.998	5.998	5.998	5.998		5.998	5.998		
	>6 to ≤12	P4 conc. <1 (oestrus)	0.001	0.816	0.231	0.122		0.757	0.811		
		P4 conc. >1 (ovulation)	1.036	3.449	1.742	1.604		2.979	3.355		
		P4 conc. ≥3 and ≤7 (midluteal period)	3.449	3.449	3.449	3.449		3.449	3.449		
	≤12	P4 conc. <1 (oestrus)	0.001	0.996	0.236	0.119		0.753	0.947		
		P4 conc. >1 (ovulation)	1.011	5.998	1.875	1.446		4.341	5.666		
		P4 conc. ≥3 and ≤7 (midluteal period)	3.449	5.998	4.723	4.723		5.870	5.971		
	>12 to ≤24	P4 conc. <1 (oestrus)	0.004	0.997	0.347	0.250		0.875	0.980		
		P4 conc. >1 (ovulation)	1.024	5.809	2.511	2.264		5.098	5.359		
		P4 conc. ≥3 and ≤7 (midluteal period)	3.007	5.809	4.035	3.718		5.316	5.675		
	≥24	P4 conc. <1 (oestrus)	0.002	0.999	0.343	0.299		0.859	0.981		
		P4 conc. >1 (ovulation)	1.002	7.996	2.831	2.154		6.027	7.898		
		P4 conc. ≥3 and ≤7 (midluteal period)	3.085	6.351	4.424	4.147		6.027	6.307		
	All ages combined	P4 conc. <1 (oestrus)	0.001	0.999	0.323	0.245		0.845	0.991		
		P4 conc. >1 (ovulation)	1.002	7.996	2.635	2.154		5.745	7.833		
		P4 conc. ≥3 and ≤7 (midluteal period)	3.007	6.351	4.264	4.037		5.960	6.267		

In the group of male cattle, of the 375 samples in which P4 was detected, 285 exceeded the CCa value determined during method validation. In the other 90 samples (24%), the P4 concentration was below 0.058 μg L^−1^ (the CCa). The highest P4 concentration (31.792 μg L^−1^) was determined in the serum sample of a male under 12 months of age. Similarly, the mean concentration of 1.170 μg L^−1^ was also the highest in this age group. Furthermore, the calculated 95^th^ and 99^th^ centiles were 1 to over 4 times higher than those for males older than 12 months. Analysing the presented results, 95% of serum samples from the group of the youngest males (<12 months) contained P4 at below 4.038 μg L^−1^ and 99% contained the hormone at below 19.227 μg L^−1^, 95% of serum samples from the group of males aged 12–24 months yielded <1.572 μg L^−1^ and 99% yielded <3.044 μg L^−1^, and the two centiles of samples from the group of males older than 24 months had <3.138 μg L^−1^ and <3.804 μg L^−1^.

In the group of female cattle, of the 679 serum samples in which P4 was determined, in 601 samples the concentration of this compound exceeded the CCa of 0.058 μg L^−1^. Only in 78 samples (11%) did the P4 concentration not reach the CCa. The highest P4 concentration (496.059 μg L^−1^) was determined in the serum sample of a female over 24 months of age. The mean serum P4 concentration was the highest in samples from cows older than 24 months and amounted to 6.348 μg L^−1^; however, similar mean concentrations were recorded for females between 12 and 24 months of age and up to 12 months of age. Based on the obtained results, 95% of serum samples from the group of the youngest females (<12 months) contained P4 at below 2.689 μg L^−1^ and 99% contained it at 49.439 μg L^−1^; 95% of serum samples from the group of females aged 12–24 months had concentrations of it below 5.108 μg L^−1^ and 99% had weaker concentrations than 23.866 μg L^−1^ and the two centiles of samples of the group of females older than 24 months were analysed for P4 at 13.667 μg L ^−1^ and 109.185 μg L^−1^.

Considering the results of P4 concentration determinations in female serum in terms of the phase of the oestrous cycle, in the entire population, the P4 concentration was less than 1 μg L^−1^ (and suggestive of oestrus) in 61% of samples. In the individual age groups considered, it was at this low level in 83% of sera from cattle up to 6 months of age, in 79% of sera from cattle between 6 and 12 months of age, in 82% in the samples from cows under 12 months of age, in 59% of the analysed material from animals between 12 and 24 months of age, and in 56% of samples taken from cows at least 24 months of age. In total, for all the sampled female cattle, the P4 concentration was in the range of 1 to 7 μg L^−1^ (suggestive of ovulation or mid-luteal period) in 33% of samples. In 13% of the sera taken from animals up to 6 months of age, in 18% of those of cows between 6 and 12 months of age, in 15% of samples obtained from cattle under 12 months of age, in 37% of material from the population between 12 and 24 months of age and in 34% of the serum samples provided by cows older than 24 months the concentration of P4 was also in this range. The detailed distribution of P4 concentration in relation to the physiological bands characteristic for defined oestrous phases is presented in the histograms in the right column of Figs 2 and 3.

**Fig. 4.**
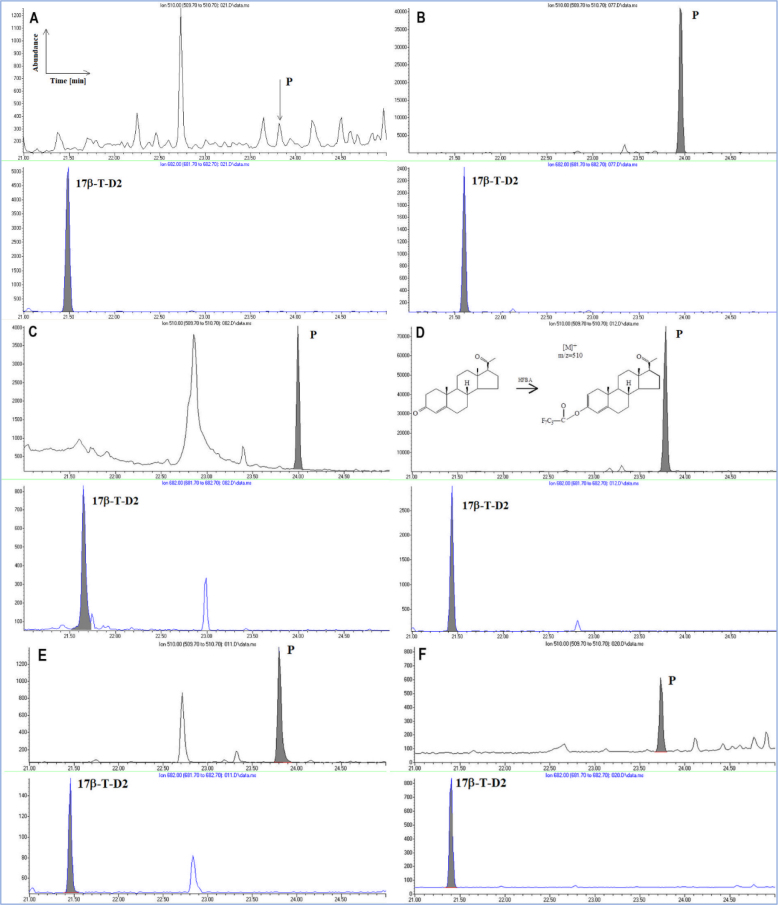
Chromatograms of A – a blank bovine serum sample; B – a serum sample spiked with progesterone (P4) at 0.500 μg L^−1^; C – a serum sample from a male bovine with P4 detected at the concentration of 1.336 μg L^−1^; D – a serum sample from a female bovine with P4 detected at the concentration of 13.548 μg L^−1^ and diagrams of structure of P4 and its heptafluorobutyric anhydride (HFBA) derivative); E – a serum sample from female bovine with P4 detected at the concentration of 4.734 μg L^−1^; F – a serum sample from female bovine with P4 detected at the concentration of 0.362 μg L^−1^. 17β-T-D2 – deuterated analogue of 17β-testosterone used as internal standard

Representative GC-MS chromatograms of blank serum samples, spiked samples and real male and female samples (illustrating sensitivity across the physiological range) with detected P4 are presented in Fig. 4.

## Discussion

Natural hormones are present in almost all biological matrices, and their interactions can make it difficult to establish universal rules for monitoring these compounds and interpreting test results. For the most analytically important substances in the group of natural sex steroid hormones, 17β-T and 17β-E2, maximum male and female age-related serum levels have been established (11, 32). These levels have been accepted as natural thresholds and are used to assess sample compliance. Progesterone, which is also a natural sex steroid hormone, is often replaced by synthetic analogues in control studies, and data on the natural levels of this compound in biological matrices of animal origin are scarce. Taking into account previous experimentation, a laboratory method optimised for 17β-E2 and 17β-T determination in bovine serum was adapted for P4. Because P4 belongs to the same group of steroid hormones as 17β-E2 and 17β-T, the adequacy of serum sample purification using the method in the procedure for these natural compounds was verified also for P4 during validation. The analytical performance confirmed the suitability of that sample purification method also for P4.

The specificity of the method for P4 was confirmed by finding no peaks in the chromatogram of blank sample that interfered with this analyte within its retention-time range. Furthermore, a proportional increase in the signal of the tested compound was noted in spiked samples. For the standard and matrix-matched calibration curves, the linear-regression parameters determined indicated a good curve fit and provided a linear response over the tested range.

For spiked serum samples, satisfactory apparent recovery values were reported for concentrations in the range of 0.500–1.000 μg L^−1^ within the reference range of 50–120% and for concentrations of 2.000 μg L^−1^ within the reference range of 70–120%. The values were good judged under the current principles of recovery assessment for test methods for veterinary drugs defined in Commission Implementing Regulation (EU) 2021/808 as the minimum trueness of quantitative methods. Good precision was obtained, also against the Regulation’s criteria, because the coefficients of variations were acceptable and less than 25% under repeatability and within-laboratory reproducibility conditions (12).

The uncertainty values determined at the spiking levels were in the range of 45–55%. The results from quality control samples and validation data were used for uncertainty estimation. The uncertainty calculations took into account the two factors that have the greatest impact on the uncertainty value, namely reproducibility and apparent recovery (17). Since the values of these validation parameters were consistent with the criteria, the uncertainty values can be considered realistic.

The CCα and CCβ parameters determined during validation reached values almost identical to that determined for 17β-T. These parameters are useful as decision value when assessing test sample compliance and the CCα has utility if the method used serves as a screening and the CCβ does if the method is a confirmatory test. According to EU Implementing Regulation 2021/808, any sample in which the concentration of the test analyte is greater than or equal to the CCα value is considered non-compliant (12). The EURL Guidance on Minimum Method Performance Requirements (MMPRs) for specific pharmacologically active substances in specific animal matrices holds the decision parameters determined during method validation for compounds with established MMPR analytical limits, to values below these limits (11). However, as a general rule, under specific methodological conditions, the detection parameter values for banned and unauthorised compounds which include hormones should be as low as reasonably achievable. It should be noted that P4 is not a recommended compound for monitoring studies in animal tissues and animal products. Furthermore, no MMPR values have been specified in the relevant EURL publication for P4 in biological matrices. The studies conducted here attach only theoretical and demonstrative meaning and importance to the CCα and CCβ parameters, as the aim of the study was to examine serum P4 levels in animals and not to test the samples for compliance. Assuming that the method was to be used for screening purposes (the only option due to the specificity of the instrumental technique), an additional experiment would be necessary to test the reliability of P4 detection in samples spiked to the determined CCα/CCβ value.

The physiological norms of progesterone concentration in cattle reproductive biology are material to validation of a screening test for it. Progesterone levels play a key role in reproductive processes. They vary depending on the stage or phase of the female’s oestrous cycle (10, 18, 23, 25, 26, 28, 29, 30). During oestrus (readiness for fertilisation and increased sexual drive, luteolytic process and lack of a functional corpus luteum), which occurs before ovulation and usually lasts 18 to 24 hours, blood P4 concentrations are below 1 μg L^−1^, but at the time of ovulation (functional corpus luteum), progesterone levels typically rise above 1 μg L^−1^, as the uterus prepares for pregnancy, and later during the mid-luteal phase, the levels typically range between 3–7 μg L^−1^. If fertilisation and pregnancy do not occur, the corpus luteum degenerates and progesterone levels drop to below 1 μg L^−1^ by the time of oestrus (10, 18, 23, 25, 26, 28, 29, 30). Progesterone levels can also be considered an indicator of egg cell quality and the uterine environment; during pregnancy, the P4 level is approximately 3–6.5 μg L^−1^ in the first trimester, rising to 3–8 μg L^−1^ by the fifth month, and its level also varies during the development of the mammary gland and the course of lactation (10, 18, 23, 25, 26, 28, 29, 30). Progesterone levels, which have their own dynamics, can also be an indicator of fertility when monitored during artificial insemination. It should be emphasised that progesterone levels, especially during pregnancy, can vary individually, and it is not possible to distinguish between pregnant and non-pregnant animals based on progesterone levels. Progesterone levels above 1 μg L^−1^ on days 17–21 indicate early pregnancy because no luteolysis is taking place. To diagnose pregnancy, it is recommended to use a pregnancy-associated glycoprotein test and ultrasound (21).

The present study is a comprehensive overview of the natural occurrence of P4 in serum samples from cattle. The test results may constitute a basis for determining national physiological concentration limits, should it be necessary to conduct P4 serum tests if the EU mandates them. Evaluation of the test results by sex and age of cattle was possible, but the impossibility of refining the analysis against richer background data may be a limitation of this study. There is no doubt that information on reproductive predisposition and reproductive status at the time of sampling would probably be particularly important in the case of females for result interpretation. Referring to the literature, many authors have presented P4 concentration measurements at various stages of the oestrous cycle in generally in dairy cattle, in pregnant cattle, in dairy cattle after calving, or in cows selected for insemination (embryo transfer), which are extremely important as clinical and reproductive status elucidation (10, 18, 23, 25, 26, 28, 29, 30).

As early as the 1970s, Gomes and Erb (18) studied P4 concentrations in ovarian venous blood plasma during the oestrous cycle in cows and other animal species using chemical assays. They observed an increase in P4 concentration from 6.5 μg L^−1^ of the 1^st^ day of the cycle to 73.2 μg L^−1^ on the 14^th^ day, followed by a decrease to 10 μg L^−1^ on the 20^th^ day of the cycle. In later years, research continued, and today it is also a subject of interest. Diaz *et al*. (10) presented P4 concentration levels in females in four breed groups of cattle which ranged from 0.5 μg L^−1^ to 5.1 μg L^−1^ in Holsteins, to 9.2 μg L^−1^ in Brahmans, to 13.7 μg L^−1^ in Caroras and to 8.8 μg L^−1^ in mixed-breed cattle, measured on the day of oestrus to give the lower end of the range and on the day of the peak of the luteal phase to give the upper end. Mekonnin *et al*. (26) reported P4 concentrations in crossbred cattle (Zebu and Holstein-Friesian) in urine, serum saliva and milk. Serum P4 concentrations in different reproductive phases in mixed dairy cows ranged from less than 1 μg L^−1^ for cows in the anoestrous state and oestrous phase to an average of less than 4 μg L^−1^ and more than 5 μg L^−1^ for cows in the dioestrous phase and pregnancy, respectively. The authors noted a close correlation between the average serum and milk P4 concentrations. In turn, Tenhagen *et al*. (28) published results of P4 measurements in post-parturient dairy cows treated with prostaglandin to induce and synchronise oestrus. It is difficult to compare our results to those in the literature without information on the treatment processes to which the animals from which the samples were taken were subjected. Terblanche *et al*. (29), like Diaz *et al*. (10), reported P4 concentrations in bovine serum during oestrus and pregnancy and after calving. On the day of oestrus, the concentration fell below 0.5 μg L^−1^, while it ranged from 4 to 7 μg L^−1^ at the peak of the luteal phase. Three weeks after artificial insemination, it was in a 3–7 μg L^−1^ range, while in the sixth to eighth weeks of pregnancy, concentrations of 3.8–6.5 μg L^−1^ and 3–7 μg L^−1^ were observed. Later in gravidity, between the second and third months of pregnancy, levels of 3.5–6.5 μg L^−1^ were measured, which increased to 3–8 μg L^−1^ from the fifth month until just before calving. Near parturition, levels of approximately 3–4 μg L^−1^ were observed up to 3 days before calving, falling to 2 μg L^−1^ on the day before calving; on the day of calving and shortly afterwards, they were below 1 μg L^−1^. Interesting results were also disseminated by Valchuk *et al*. (30), who measured P4 concentrations in cows on the seventh day after oestrus and after embryo transfer, dividing the animals into three groups according to P4 concentrations below 2.5 μg L^−1^, between 2 and 5 μg L^−1^ and above 5 μg L^−1^.

Analysing the results of our study, without knowing the reproductive status of the females, we can see correlations with the results of Diaz *et al*. (10) and those of Terblanche *et al*. (29). In our experiment, in the age group below 12 months, 97% of the results for females ranged from 0.001 to 6.185 μg L^−1^, 94% for females aged 12 to 24 months ranged from 0.004 to 4.765 μg L^−1^, while 97% of the concentrations for cattle over 24 months of age ranged from 0.002 to 24.805 μg L^−1^. Based on literature data, a rough interpretation of the female cattle P4 assay results in our study can be made. The results obtained for the entire female population indicate that the 61% with P4 concentrations below 1 μg L^−1^, regardless of their intended use, were individuals in the peri-oestrous phase of their reproductive cycle. The 33% of females with P4 concentrations ranging from 1 to 7 μg L^−1^ were indicated to be post-ovulatory individuals in the luteal phase of their reproductive cycle. However, it should be assumed that these were not pregnant females, as the monitoring sampling strategy for hormone testing, especially for natural hormones (17β-E2 and 17β-T), excludes such individuals. However, over 94% of results for the studied females are consistent with accepted standards for bovine reproductive physiology published in the literature. The 6% result proportion in which the P4 concentration exceeded 8 μg L^−1^, comprises data difficult to interpret, knowing the assumptions of the monitoring programme; however, it is possible to conclude irregularities in the functioning of the hormonal system of these animals, irregularities in the treatment of these animals or, ultimately, unreported pregnancies and phenomena of their courses and consequences.

The results for male cattle cannot be compared as there are no literature data on serum P4 levels for this sex, most likely because the sex hormone testosterone plays the key role in male reproduction. In males, progesterone is produced by the adrenal glands, which means that its presence in the blood (serum) is physiological and, as a rule, the concentration of this hormone does not reach high levels. Its level may be important in the examination of adrenal gland disorders, but it is not assessed in cattle breeding practice. Its production in the body can be linked to the exposure of animals to stressful situations, such as those that occur in slaughterhouses prior to slaughter, and can be associated with the presence of P4 in post-slaughter serum.

Many researchers have shown results of P4 determination in edible matrices of animal origin, such as milk and eggs, as well as in the environment or antlers (1, 24, 33, 34). Some laboratories which determined acetylgestagens residues in kidney fat tissue, muscles, and feed as is recommended, also conducted research on synthetic P4 derivatives, but focused on plasma, for example (6, 9, 27). In our laboratory, we also took advantage of the availability of samples from other matrices (muscle tissue, kidney fat tissue and milk), and determined P4 levels in them over a similar time range to that of the serum samples; the results of these studies are currently being summarised.

Tests to monitor residues of hazardous substances are extremely important from the point of view of food safety and the health of consumers of food of animal origin (13, 14, 16). If progesterone is included among the natural steroid sex hormones which are deemed hazardous at or above certain content levels in food of animal origin, monitoring tests will need to encompass this substance. This will also be crucial from the perspective of trade and exchange of goods, not only within the EU, given that in some countries around the world the use of hormones in food-producing animals is not prohibited.

## Conclusion

Increasing productivity in cattle farming, greater availability of veterinary medicinal products, changes in recommendations for monitoring of hormone residues in animals and animal-derived foods and consumer demand are prompting research to assess the risks posed by these hazardous substances. The serum tests detailed in this article will serve as a basis for compiling other test results available now and in the future and for creating a statistical database on natural hormone levels, including P4 in other animal-derived matrices, especially those intended for consumption.
